# Blowing in the wind? Testing the effect of weather on the spatial distribution of crime using Generalized Additive Models

**DOI:** 10.1186/s40163-022-00171-2

**Published:** 2022-10-01

**Authors:** Rannveig Hart, Willy Pedersen, Torbjørn Skardhamar

**Affiliations:** 1 Norwegian Institute of Public Health, Skøyen, P.O. Box 222, 0213 Oslo, Norway; 2 Department of Sociology and Human Geography, University of Oslo, Oslo, Norway

## Abstract

Oslo, the capital of Norway, is situated in a North European cool climate zone. We investigate the effect of weather on the overall level of crime in the city, as well as the impact of different aspects of weather (temperature, wind speed, precipitation) on the spatial distribution of crime, net of both total level of crime, time of day and seasonality. Geocoded locations of criminal offences were combined with data on temperature, wind speed, and precipitation. Generalized Additive Models (GAMs) allowed us to map level of and the spatial distribution of crime, and how it was impacted by weather, in a more robust manner than in previous studies. There was slightly more crime in pleasurable weather (i.e. low precipitation and wind speed and high temperatures). However, neither temperature, precipitation nor wind speed impacted the spatial distribution of crime in the city.

**Supplementary Information:**

The online version contains supplementary material available at 10.1186/s40163-022-00171-2.

## Introduction

A large and growing literature regards the spatial distribution of crime. Knowledge about *where* crime takes place is of interest to the public and the police, and may also cast light on more general patterns of action and interaction within cities, furthering our understanding of the social dynamics underlying crime patterns (see e.g. Andresen, 2009; Weisburd et al., 2012). Previous studies have shown that season (Andresen & Malleson, 2013; Quick et al., 2019) as well as weekday (Andresen & Malleson, 2015) may influence where crime takes place. Seasonal weather variation has been suggested as an explanation of these seasonal location shifts. This possible explanation has received little empirical scrutiny, in spite of a large literature documenting effects of weather on overall crime counts (Cohn, 1990; Cohn & Rotton, 1997; Ranson, 2014).

In this paper, we estimate the effect of weather on the spatial distribution of crime. The contribution of the paper is twofold. First, we increase the understanding of seasonal and weather-driven effects on crime by including a spatial perspective on weather and crime. Second, we contribute to the general methodological literature of spatial distribution of crime, drawing on methods established and applied in other research areas. Analytical tools used in this literature have tended to either excel at describing and testing local differences, by means of e.g. Spatial Point Pattern Test (Andersen, 2009), quad plots (Corcoran et al., 2011) or by incorporating covariates and testing general local dependency (e.g. spatio-temporal regression, see Quick et al., 2019). As a tool that serves both these purposes, we employ Generalized Additive Models (GAMs) to model the spatial surfaces, as extensively used in spatial modelling in other fields (Wood, 2017). Simple comparisons of model fit can shed light on whether two spatial surfaces—for instance the distribution of crime on rainy vs. not rainy days—are statistically different. Mapped predictions give an intuitive overview of the magnitude and location of effects. The models can include covariates, and the importance of these covariates can be assessed both by comparing model fit, and by comparing the predicted spatial surfaces with and without covariates.

The flexibility of the GAM model for spatial analysis allows us to cast new light on how weather impacts the spatial distribution of crime. We describe the spatial distribution of crime in Oslo and explore the effect of weather on the spatial distribution of crime. Our multivariate georeferenced framework allows for more detailed comparisons than have been possible in previous studies, estimating effect of each weather type net of other weather types, time of day and seasonality.

### Review of the literature on weather and the spatial distribution of crime

There is a long research tradition documenting that weather can affect crime, although the nature of the relationship may vary between contexts (Cohn, 1990). Using a 30-year panel of criminal activity in USA, Ranson (2014) found a strong positive effect of increasing temperature on nine major categories of crime. Cruz et al. (2020) found that outdoor violence in Ohio, US, increased in high temperatures. Ceccato (2005) found that homicides increased in Sao Paulo, Brazil, with higher temperatures, and Goin et al. (2017) showed that a Californian drought had criminogenic effects. A review in *Science* (Hsiang & Kopp, 2017) echoes this worry with respect to climate, hypothesizing that climate changes may impact a variety of domains, including crime.

The criminology of place has documented that “hot spots” account for a large proportion of crime in cities and that such patterns seem to be relatively stable over time (Hipp, 2016; Weisburd et al., 2012; Weisburd, 2015: p. 149). This suggests that police work should be geographically focused, and studies of policing effectiveness support such strategies (Weisburd & Eck, 2004). For instance, neighborhoods with many alcohol outlets have been linked to high rates of crime (Gorman et al., 2001). Drug scenes where illegal drugs may be used openly or sold are also associated with violence and burglary (Fast et al., 2017; Sandberg & Pedersen, 2009). Gerell et al. (2021) finds that gun violence in Swedish cities is strongly concentrated in deprived areas with open drug markets, and Guldåker et al (2021) find that a majority of the most crime-exposed urban areas overlap with socially vulnerable areas in Sweden. For Oslo, Allvin (2019) finds strong spatial patterns in burglary and vehicle theft.

Weather changes do not necessarily affect overall crime rates; instead, it may rather lead to crimes being committed at alternative locations (Quick et al. 2019). A small number of studies have tested if weather affects the spatial distribution of crime, contrasted with a null hypothesis of no dislocation effects of weather. Brunsdon et al. (2009) studied the effect of weather on the spatial distribution of police-related incidents in an urban UK area. They used a comap approach (Brunsdon, 2001), in which spatial kernel densities of crime are estimated based on four weather characteristics, controlling for time-of-day effects. Temperature and humidity affected the spatial distribution of crime significantly in both summer and winter, whereas there were no effects of precipitation and wind. Using the Spatial Point Pattern Test (SPPT) (Andresen, 2009), Schutte and Breetzke (2018) found differences in the spatial distribution of violent crime and property crime, but not sex crimes, by temperature and rainfall in Tshwane, South Africa. Due to climatic and other contextual differences, the Brunsdon study from UK would be expected to be the most similar to the Norwegian context.

Furthermore, a small number of previous studies have explored the qualitative nature of the spatial dislocation effects, i.e. not only *if* weather has an effect, but also *how* spatial crime patterns change with weather. Using a spatio-temporal regression model, Quick et al. (2019) found that, in warm seasons, crime rates in Ontario, Canada, were higher in areas dominated by parks, whereas in colder seasons, crime rates were higher in areas with nightlife. Corcoran et al. (2011) found some evidence that the increase in city fires on warm days is greater in poor neighborhoods. Ceccato (2005) analyzed location data on homicides in Sao Paulo, Brazil, using a clustering technique. Their findings indicated that increases in the level of crime tends to go together with the diffusion of crime in space (Ceccato, 2005). Castle and Kovacs (2021) finds that crime in a small Canadian city is more spatially dispersed in summer than winter.

A related literature has explored seasonal variation in the spatial distribution of crime (see e.g. Ceccato, 2005; Haberman et al., 2018; Harries et al., 1984; Morken & Linaker, 2000), and it has been suggested weather as a driver of these seasonal variations (Andresen & Malleson, 2013). Understanding how weather impacts the spatial distribution of crime casts light on one of the possible drivers of the seasonal variation in the spatial distribution of crime.

### Theoretical framework and research question

Our theoretical point of departure is the broad routine activity framework, suggesting that individuals make decisions based on rational considerations of the costs and benefits of alternative choices (Becker, 1968; Cohen & Felson, 1979; Cornish and Clarke, 2014). As an extreme example, lockdowns to curb the spread of COVID-19 may radically alter movement patterns and thus the spatial patterns in crime (Dewinter et al., 2021).

Research on the effect of weather on crime and the spatial distribution of crime share an emphasis on criminogenic *contexts:* crime happens when and where potential offenders and targets meet (Carleton et al., 2016; Kelly & Kelly, 2014). Weather influences where people stay and what they do, and thereby the likelihood that one can commit a crime and not get caught, i.e. the criminogenic opportunities (Agnew, 2012; Jacob et al., 2007; Rotton & Cohn, 2003): If people may leave their homes to enjoy good weather, public places like parks and recreational spaces may be filled with potential targets on a warm and dry day, but not on a cold and rainy day. Potential offenders may anticipate this, and e.g. more often seek for targets in parks on a warm and sunny days than on cold and rainy days. Alternatively, bad weather may facilitate crime by discouraging both the availability and capability of guardians (Tompson & Bowers, 2013). As such, there is no need to restrict the discussion to temperature and heat, as precipitation and fog, for example, also might affect behavioral and crime patterns (Tompson & Bowers, 2015).

In this paper, we will explore if weather impacts the spatial distribution of crime in Oslo using a GAM model. We will explore if they are concentrated in known areas for outdoors recreation, and if dislocation effects (if any) take the form of diffusion effects, increasing crime counts more in areas where they were originally low.

### The context of Oslo

**Fig. 1:**
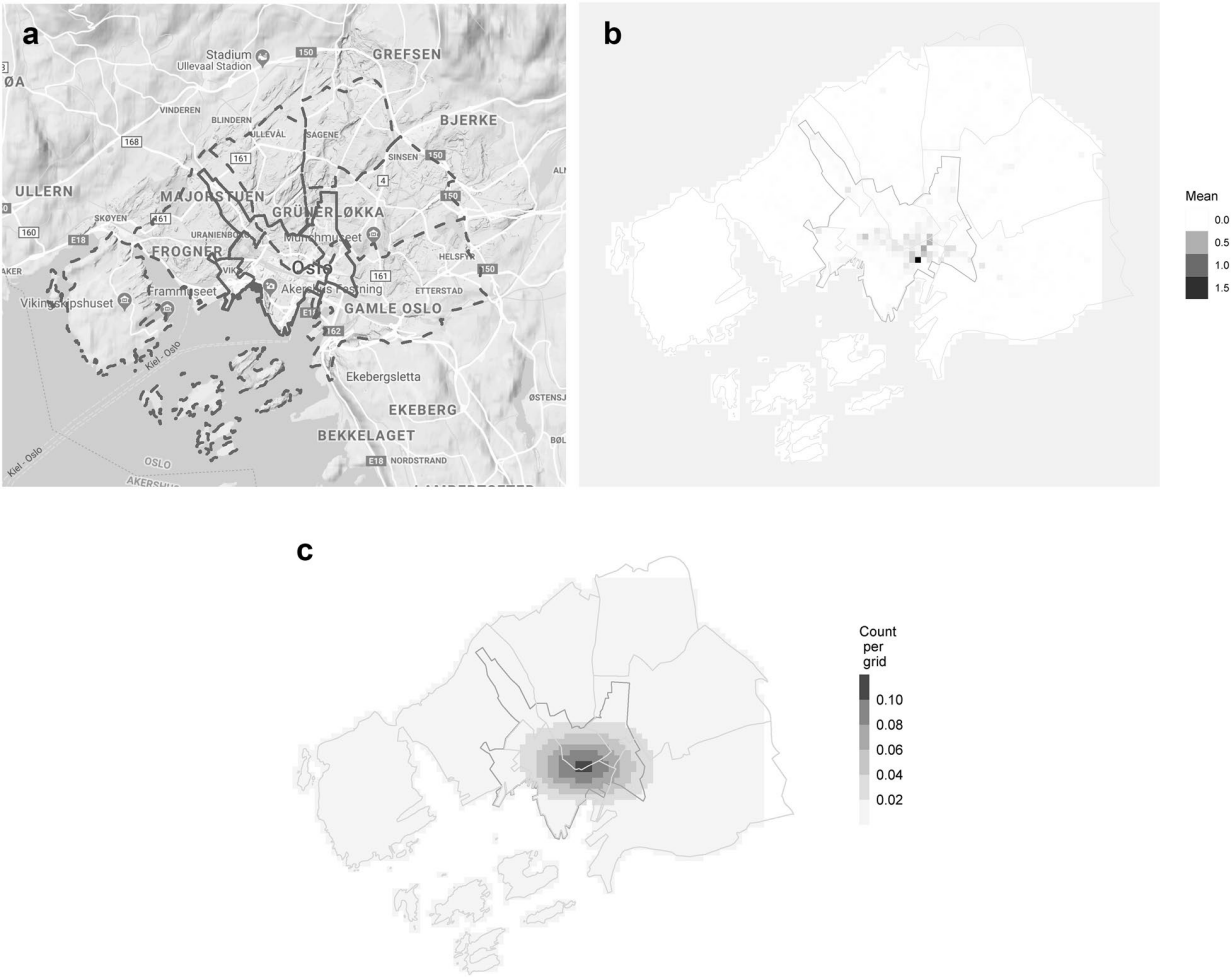
Spatial descriptives. **a** Map of the central city districts of Oslo. Map: © Google. Dashed (light gray) lines give city district borders, full (dark gray) lines give “functional city center” borders. **b** Descriptive plot of the spatial distribution of crime. Mean crime count per 6-h slot and 100-m grid. **c** Basic spatial distribution of crime all counts per 6 h-slot and 100 m gird. Prediction from basic GAM (Model 1, Table 2). The model includes fixed terms for season dummies, time of day dummies, and linear terms for temperature, rain, and wind. See Table 2 for fixed term estimates, significance of smoothers, and model fit statistics

With about 600,000 inhabitants, Oslo is a small European capital. Although crime rates are low by international standards, Oslo is by far the most criminogenic area in Norway.^1^ Crime linked to heavy drinking has been a cause of public concern in Oslo (Rossow & Norstrom, 2012; Skardhamar et al., 2016). According to the police, the “functional city center” (marked by full lines on the map of Fig. 1a) has the busiest shopping districts and most places to buy alcohol. A western “arm” goes through the busy shopping and nightlife area around Bogstadveien, and an the eastern “arm” goes towards the gentrified restaurant and nightlife area of Grünerløkka. Crime has also been linked to the two illegal drug distribution scenes in Oslo. The hard drug distribution scene in an area near the Central Railway Station is the most criminogenic and most heavily policed part of Oslo (Sandberg & Pedersen, 2008). Cannabis is illegal in Norway, and cannabis dealing has been taking place in a larger area along a river in Central East Oslo (Sandberg & Pedersen, 2009).

## Methods

### Data

Our data set summarizes crime counts in 6-h slots and 100 m wide quadratic grids. Observations start on July 27, 2010 (the first date of complete hourly weather data), and we obtained crime data from police registers (STRASAK, “Politiets straffesaksystem”), including all crimes reported in the municipality of Oslo from July 2010 through December 2014. For each reported incident, we obtained data about its location (aggregated to 100-m grids), time of occurrence, and type, as well as when the report was filed.

Time of occurrence is recorded by the police as exact time if known by the one who reported the crime, or the time of intervention if detected by the police themselves. The data include a start and stop date-time stamp for event time. We use the start time as event time, and include only events with intervals less than 4 h (i.e. where event time is known with reasonable precision). The accuracy is variable given the nature of the data, but we consider that to be of minor importance since we are rounding to 6-h slots: Even with small imprecisions measurement, most events will be classified in the correct time slot. There is a tendency for events with uncertain times to be coded as 00 (midnight). Most events with unknown time will have taken place at nighttime and are thus classified into the correct slot. A small number of daytime events with missing time assigned 00 could introduce a small measurement error and hence a weak dilution of effects.^2^

Our data set is restricted to property offences, drug-crimes, and violent crimes, such crime types that frequently occurs outside of private homes.^3^ Property offences are crimes for profit, and include both robberies of persons as well as theft from shops, houses and other public places as well as theft of bikes, or vehicles, but not fraud, embezzlement etc. that do not dependent on specific location. Violence includes all interpersonal violence and threats, except violence in family relations and sexual crimes. Drug crimes include all drug crimes, but is dominated by dealing, use and possession of illegal drugs.^4^ We also estimated our models separately for these three categories of crime.

**Fig. 2:**
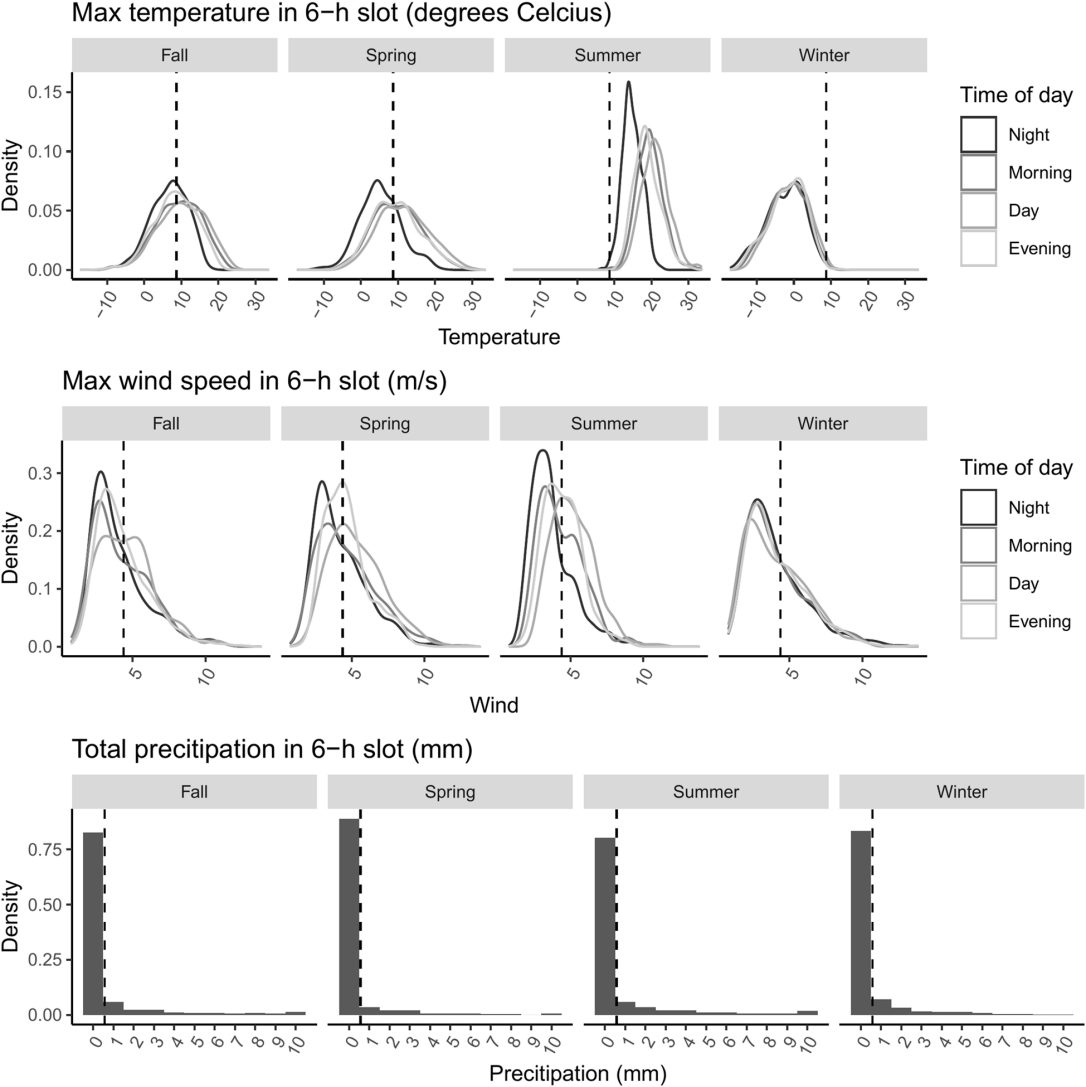
Summary statistics for the weather predictors: temperature, wind speed, and precipitation. Measured in 6-h slots and displayed separately by time of day and season. Dotted lines give the unconditional mean of the plotted variable

Crimes geocoded at locations outside of the six most central districts of Oslo were excluded for these analyses (see Fig. 1a and b).^5^ Crime counts were aggregated in 6-h slots (00:00–05:59, 06:00–11:59, 12:00–17:59, 18:00–23:59) separately for each of the three crime types and jointly for all three types. The intervals are meant to separate reasonable activity periods in Oslo. Nightlife where licensed premises closes at the latest 03:00, with crimes at the following hours also relating to outdoors gathering after closing time, are captured by the first interval. The next two intervals capture normal office hours, and then the evening constitutes the final interval. We split the year into four seasons: Fall (September–November), Winter (December–February), Spring (March–May) and summer (June–August). Summary statistics for weather and crime counts are shown in Table 1. More detailed descriptives for weather by time of day and season are given in Fig. 2.

**Table 1:** Summary statistics

All seasons	Mean	Min	Max	Median	Max. total
Crime count per grid	0.01	0.00	20.00	0.00	11.804
Precipitation (mm)	0.62	0.00	58.70	0.00	
Temperature (degrees Celcius)	8.69	− 17.00	33.40	8.80	
Wind speed (m/s)	4.38	0.80	13.80	4.00	
Fall					
Crime count per grid	0.01	0.00	17.00	0.00	3.400
Precipitation (mm)	0.70	0.00	20.80	0.00	
Temperature (degrees Celcius)	8.59	− 11.40	23.30	8.70	
Wind speed (m/s)	4.31	1.00	12.40	3.90	
Spring					
Crime count per grid	0.01	0.00	18.00	0.00	2.963
Precipitation (mm)	0.39	0.00	17.50	0.00	
Temperature (degrees Celcius)	9.02	− 11.80	29.80	8.60	
Wind speed (m/s)	4.73	1.40	12.80	4.40	
Summer					
Crime count per grid	0.01	0.00	20.00	0.00	2.538
Precipitation (mm)	0.91	0.00	58.70	0.00	
Temperature (degrees Celcius)	18.57	6.70	33.40	18.40	
Wind speed (m/s)	4.42	1.40	11.30	4.20	
Winter					
Crime count per grid	0.01	0.00	17.00	0.00	2.903
Precipitation (mm)	0.45	0.00	11.90	0.00	
Temperature (degrees Celcius)	− 1.69	− 17.00	12.00	− 1.10	
Wind speed (m/s)	4.08	0.80	13.80	3.60	

Explanatory variables and outcome variables

Jointly for all seasons and separately by season

Observations are made per grid cell, within 6 h slots (column 1–4) or throughout the observation period (column 5)

The Norwegian Meteorological Institute makes data on weather publicly available through an API service.^6^ We aggregated hourly information on precipitation, wind speed and temperature into the same 6-h slots. For each slot, we calculated maximum wind speed, maximum temperature, and the sum of precipitation (rain/snow). Missing data were rare and ignored in the calculations.^7^

1619 unique dates with 6,475 time slots in total, divided into 3197 grid cells, which yielded 20,700,575 observations.^8^ Weather data were linked to information on weather conditions, specific to the 6-h slot. As weather is truly exogenous to crime, reverse causality was not a concern, and subsequent predictors merely constituted noise. Inclusion of lagged temperature predictors did not improve the model (results available upon request). Descriptive statistics for the spatial distribution of crime variables are shown in Fig. 1b.

### Modeling effects of weather on the spatial distribution of crime

To estimate the effect of weather on the spatial distribution of crime, we describe the differences between two (or more) spatial surfaces and test if these differences are statistically significant. In the literature on the spatial distribution of crime, several techniques serve the first purpose [e.g. Andersen’s Spatial Point Pattern Test (2009) or quad plots (Corcoran et al., 2011), or regression techniques that handles spatially correlated observations (Ward and Gleditch, 2018)]. To assess whether the overall spatial distribution changes, we need to compare spatial densities of crime. Kernel density estimates is commonly used to this end, but comparisons across weather conditions, and the handling of confounders using control variables, are done more efficiently in a regression framework.

In this study, we propose that Generalized Additive Models (GAMs) as an accessible and efficient tool in estimating spatial surfaces with covariates. The technique is widely applied in other fields, based on a regression framework known to most analysts and unifies the goals of intuitive mapping of results with inclusion on control variables. Coordinates are included as a semiparametric smoothing spline (tensor product smooth) that captures the spatial surface. Alternative smoothers—thin plate regression and p-smoothers—do not change our results. We conduct our estimations in R (R Core Team, 2018) using the *bam*-function for very large datasets (Wood, 2017).

In a descriptive first step, we describe the spatial distribution of crime in Oslo using predictions from a basic GAM. The basic spatial regression model (Model 1) then becomes

1 $$log\left({\lambda }_{it}\right)=\alpha +s\left({lat}_{it}, {lon}_{it}\right)+\gamma {W}_{t}+\zeta +\eta$$

where ${\lambda }_{it}$ is the number of crimes in grid cell *i* at time *t*, and *s* denotes a smoothing surface over the coordinates.^9^ The vector ${W}_{t}$ includes continuous measures of precipitation, wind and temperature. Their coefficients will give the effect on weather on crime in Oslo, irrespective of location. Fixed effects for season,$\zeta$, and time of day, $\eta$, net out the effect of season and time of day. This allows us to capture the effect of within-season variation weather, rather than correlated seasonal/time-of-day changes in both weather and crime (see Fig. 2 for seasonal and time-of-day variation in weather in our sample). In sensitivity analysis, we explore the importance of these controls for our results.

In our second step, we estimate effects of weather on the spatial distribution of crime. To do this, the weather variables are grouped into factors: a dummy variable taking 1 if there is any precipitation (otherwise 0), and terciles for wind speed and temperature. For each weather characteristic, we estimated a separate spatial surface based on the value of the grouped variable.^10^ The estimations were done separately for precipitation (Model 2a), temperature (Model 2b), and wind speed (Model 2c). Letting W_j_F be the factorized interaction variable for weather characteristic j, the model then becomes:

2 $$log\left({\lambda }_{it}\right)=\alpha +s\left(\left({lat}_{it}, {lon}_{it}\right)|{WjF}_{it}=w\right)+\gamma {W}_{t}+\zeta +\eta$$

Note that while the interaction variable is discretized, continuous variables for wind, precipitation and temperature are still included in all models in the vector ${W}_{t}$. As such, we measure the impact of the interaction variable on the spatial distribution of crime, holding the impact of all weather variables on the level of crime constant.

### Presentation of results

We show exponentiated regression coefficients (fixed term estimates) (Table 2), which pertain to Incidence Rate Ratios as our GAM uses a Poisson link function. For smoothers, we show estimated degrees of freedom (EDF). Fit statistics (adjusted *R*^2^ and deviance explained) comparing each interaction model to the basic model were used to assess the importance of the effects of weather on the spatial distribution of crime (Wood, 2017). We illustrate spatial results graphically through maps showing grid-specific predicted crime counts.^11^ Our model does not give point estimates for the interaction terms. Rather, we compare spatial surfaces by calculating the difference in predictions for each grid cell, as shown by this example for precipitation:

**Table 2:** Results from Generalized Additive Models for crime counts in 100-m grids and 6-h slots

Fixed term estimates	Basic				Precipitation				Temperature				Wind			
	Est	(C.I.)			Est	(C.I.)			Est	(C.I.)			Est	(C.I.)		
Intercept	0.000	(0.000–0.000)***			0.000	(0.000–0.000)***			0.000	(0.000–0.000)***			0.000	(0.000–0.000)***		
Temperature (degrees Celsius)	1.002	(1.001–1.003)***			1.002	(1.001–1.003)***			0.999	(0.998–1.001)			1.002	(1.001–1.003)***		
Precipitation (mm)	0.995	(0.993–0.998)**			0.996	(0.993–0.999)*			0.996	(0.993–0.998)**			0.995	(0.993–0.998)**		
Wind speed (m/s)	0.994	(0.991–0.997)***			0.994	(0.991–0.997)***			0.994	(0.991–0.997)***			0.993	(0.987–0.999)*		
Time of day (ref = 0–6)																
6–12	0.365	(0.358–0.372)***			0.365	(0.358–0.372)***			0.364	(0.357–0.371)***			0.365	(0.358–0.372)***		
12–18	1.000	(0.985–1.015)			1.000	(0.985–1.015)			0.997	(0.983–1.012)			1.000	(0.985–1.015)		
18–24	0.860	(0.848–0.873)***			0.860	(0.848–0.873)***			0.858	(0.846–0.871)***			0.860	(0.848–0.873)***		
Season (ref = Fall)																
Spring	1.024	(1.008–1.04)**			1.024	(1.008–1.039)**			1.024	(1.009–1.04)**			1.024	(1.008–1.04)**		
Summer	0.911	(0.894–0.927)***			0.911	(0.894–0.928)***			0.901	(0.883–0.918)***			0.910	(0.894–0.927)***		
Winter	0.907	(0.890–0.924)***			0.907	(0.890–0.925)***			0.904	(0.886–0.921)***			0.907	(0.890–0.924)***		
**Smoothing splines**			**EDF**				**EDF**				**EDF**				**EDF**	
Baseline surface			23.69***													
No rain							23.90***									
Rain							23.77***									
Surface T3											23.81***				23.85***	
Surface T2											23.76***				23.73***	
Surface T1											23.80***				23.68***	
Model fit																
R2	0.025				0.025				0.025				0.025			
Dev.expl	0.232				0.232				0.233				0.233			
N	20,700,575				20,700,575				20,700,575				20,700,575			

The outcome is grid-specific crime counts

The basic model includes a spatial surface (as a semiparametric smoothing spline), as well as three weather characteristics, and sets of dummies for time of day and season

In the weather specific models, the spatial surface is additionally allowed to vary with one discretized weather characteristic (rain, temperature or wind)

***p < 0.001; **p < 0.01; *p < 0.05

3 $${Diff}_{grid=G}={Pred}_{grid=G, Percip=1}-{Pred}_{grid=G, Percip=0}$$

**Fig. 3:**
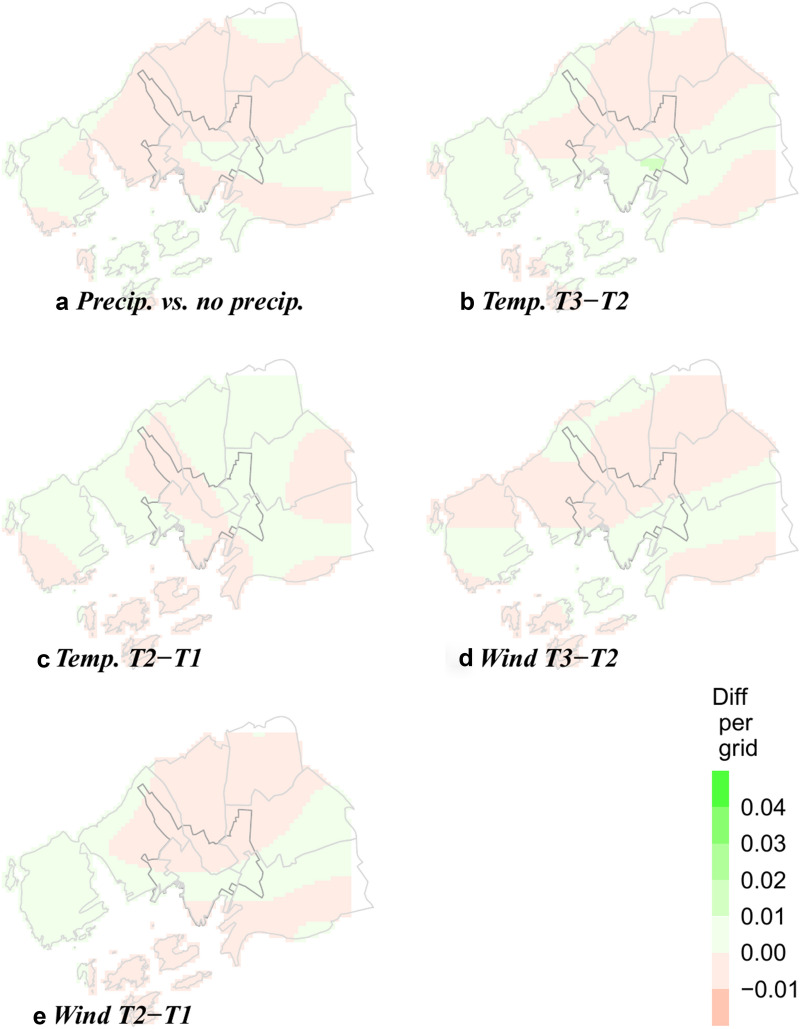
The effect of weather on the spatial distribution of crime. Differences in predictions from models with controls for time of day and season (Models 2a–c, Table 2)

These differences, presented in Fig. 3 and Additional file 1: Figures S1–S4, bears resemblance to the quad plots (Corcoran et al., 2011).

## Results

Descriptive statistics for the spatial distribution of crime—mean crime count per 6 h period by 100-m grid—are shown in Table 1 and Fig. 1b. The maximum number of crimes observed in an area this size over 6 h is 20, and the mean is 0.01. There some variation by season in the maximum crime count, with the highest counts observed in summer, and the lowest maximum counts observed in fall and winter. Column 5 shows the maximum total crime counts observed in a single grid for the full observation period. This reveals a peak of 11.804 incidents in total in one grid. The same city center cell has the maximum count in all seasons.

Figure 1b shows the average crime counts for 6 h time slots by grid cell. The six central city districts and the “functional city center” (the areas with shopping districts as well as night life in Oslo, see above) are indicated on the descriptive map by light and dark full lines, respectively. Allowing the mean crime counts to vary by grid reveals substantial geographical variation: In the area around the central station, a major trade area as well as an open scene drug use and injection, the average mean crime count per time slot is above 1. Other areas, mainly in the southern islands and the northernmost part of the city center, have a mean of zero (and hence no variation in the outcome variable).

The fixed term estimates from our basic model also reveal the effect of weather on the overall level of crime in Oslo (Model 1, Table 2). We see that temperature has a weak positive effect, with one degree higher temperature increasing crime counts with a factor of 1.002. A one millimeter increase in precipitation, on the other hand, reduces crime counts with a factor of 0.995, while a one unit increase in wind speed reduces crime counts with comparable 0.994. This pertains to a general pattern where crime counts are higher in what is generally perceived as pleasurable weather (in the Nordic climate)—low precipitation and lower wind speed, and high temperature. Such a pattern is broadly consistent with routine activity theory, suggesting that crime counts increase when weather facilitates people leaving their homes.

Compared to the reference category (Fall), we see that crime counts increase with a factor of 1.024 in Spring and are reduced with a factor of 0.911 in summer (holding weather constant). Furthermore, crime counts are highest at night (0–6 am) and midday (12–18), and lowest at early daytime (6–12). These patterns of season and time of day are largely stable when we allowed the spatial distribution of crime to vary with weather in Model 2.

### The effect of weather on the spatial distribution of crime

We now turn to our main research question, estimating the effect of weather on the spatial distribution of crime. Results are obtained from spatial interaction models (Eq. 2), and all regression coefficients are displayed in Table 2. When the spatial surface is allowed to vary by precipitation (Model 2a), temperature (Model 2b) or wind speed (Model 2b), R^2^ and deviance explained are virtually unchanged relative to the basic model (Model 1), indicating no model improvement.

Figure 3a show the grid and time specific *differences* in crime rates on days with rain/snow versus no precipitation. Effects of precipitation minuscule: the largest effect (0,002) is equivalent to an increase of one crime incident per 114 days.^12^ The same holds for differences by temperature in terciles (Fig. 3b and c) and wind in terciles (Fig. 3d and e). Taken together with no improvement in model fit, we conclude that these three dimensions of weather have no systematic effects on the location of crime in Oslo. In line with previous studies, we find that weather impacts the level of crime throughout Oslo. Our results show that this impact is evenly geographically distributed.

### Sensitivity tests

Our main model lumps together incidences from the three main categories: drug, violence, and property crime. To explore any differential patterns by crime type, we ran separate analysis for these three categories. Differences between predictions are shown in the Additional file 1: Fig. S1 (property), Fig. S2 (drug related crime) and Fig. S3 (violent crime), see Additional file 1: Table S1 for coefficients and model fit. The predicted differences by weather remain small and seemingly haphazard also when data are disaggregated by crime type. As such, the collapsed measure we use in our main models does not hide strong effects of weather on some types of crime.

The subsample models further show that the effect of weather on crime counts varies by crime type (Additional file 1: Table S1). Drug related crime is most strongly influenced by weather, with increasing counts when temperature is high, and lower counts in wind speed and precipitation (though the latter is not statistically significant). Effects for property crime goes in the same direction. Higher crime counts give more power in the property sample, and while the point estimate for wind speed is closer to 1 here than in the drug sample, it is statistically significant for property crime only. Violent crime increases in temperature, but unlike the other crime types, it is not influenced by precipitation or wind speed.

Finally, Additional file 1: Fig. S4 and Table S2 shows results from a model omitting season fixed effects. A comparison with the main results in Fig. 2 shows that omitting the fixed effects for season and time of day have little bearing on the (absence of) effect of weather on the spatial distribution of crime. We also tested whether results were robust to an alternative grouping of time slots (03:00–08:59, 09:00–14:59, 15:00–20:59, 21:00–02:59), again finding comparable results (Additional file 1: Fig. S5).

## Discussion

Our results showed that variations in the weather in Oslo do not influence the spatial distribution of crime in any systematic or substantial way. In contrast, profound dislocation effects for weather variations are found in contexts such as Brazil (Ceccato, 2005) and South Africa (Schultze & Breetzke, 2018). Norway has a cool climate, and to the extent that only very high temperatures influence aggression, it may not get warm enough to trigger aggressive behavior in larger groups. However, spatial dislocation effects were identified also in contexts comparable to the Norwegian in terms of both climate and social structures, such as Ontario, Canada (Quick et al., 2019) and UK (Brunsdon et al., 2009). One explanation that fits with previous research is that in particular the area linked to illegal drug use and distribution is a relatively stable hotspot, also across weather types.

Our results demonstrate the usefulness of General Additive Models (GAMs) in modelling, comparing and visualizing spatial surfaces, with the inclusion of covariates. We modelled separate spatial surfaces for crime by precipitation, temperature and wind speed. The use of a multivariate model allowed us to present these spatial effects net of weather effects on the level of crime. Applying smoothers allowed us to both retain test strength and avoid pitfalls of multiple testing, as compared to methods that apply a series of local statistical tests (e.g. Andresen, 2009). Simultaneously, results were mappable and provided a smoothed, yet accurate, representation on the spatial pattern of crime in Oslo, Norway. These methodological differences may also explain why our results diverge from findings from the UK and Canada. Our study more efficiently controls for effects on the level of crime. While our smoothing strategy for modelling spatial variation may make it more difficult to find small local (grid-specific) effects, our model correctly identified known hot spots, easing this concern. The smoothing approach reduces the risk of false positives that emerge when multiple local tests are applied. In other words, it better allows us to identify broad patterns of spatial shifts—or conclude that there are no substantial changes.

Previous studies have shown seasonal variation in the spatial distribution of crime in Canada (Andresen & Malleson, 2013; Quick et al., 2019) and the UK (Brunsdon et al., 2009) and variation in weather has been suggested as an explanation (Andresen & Malleson, 2013; Ceccato, 2005). Our multivariate mapping approach allowed us to test directly if weather impact the spatial distribution crime. In the Norwegian context, we find no evidence of this. To the extent that our findings are valid also in comparable contexts, changes in routine activities not related to day-to-day changes in weather explain the seasonal spatial patterns in crime detected in other studies.

While not the main purpose of our study, our findings also cast light on the effect of weather on (the level of) crime counts in Oslo. Although previous studies have tended to find an effect of warm weather, our results suggest this effect is, at best, modest in our northern climate. The weather affected crime by increasing counts moderately when the weather was better (higher temperatures) and decreasing them somewhat when the weather was worse (more precipitation and wind). The size of the significant effects were consistently small. The finding that crime counts were higher in good weather fits with expectations from both routine activity theory (Cohen & Felson, 1979) and the rational choice perspective of crime (Becker, 1968; Cornish and Clarke, 2014). In better weather, both targets and offenders are more likely to be outside and thus more likely to meet. The notion that worse weather may increase crime by incapacitating capable guardians (Tompson & Bowers, 2013) was not supported by our data.

The spatial models showed that the location of drug related crime was not influenced by weather. However, counts of drug related crime were more sensitive to weather in our sample than property crime and violent crime. One potential explanation for this is that drug crime incidents, to a larger extent than property crime and violent crime, were based on “proactive” (rather than “reactive”) policing (Ashby & Tompson, 2017; Black and Reiss, 1970). In other words, the number of drug crimes was likely the most sensitive type of crime to discretionary police activity (stop and search), as such crimes are known to be influenced by contextual factors such as the day of the week and large events (Ashby & Tompson, 2017), and weather (Ashby & Tompson, 2018). Thus, the effects on drug crime incidents may be evidence of more intense policing when the weather is good. Combined with the absence of dislocation effects, our results suggest that there is more intense policing towards drug related crimes in good weather, while where this policing takes place is unaffected.

Some limitations should be noted. First, to assess how an individual’s risk of victimization varies in space, our models should ideally have accounted for the population at risk (Gerell, 2018). Number of residents in an area will unfortunately be a misleading estimate of the population at risk, as some very central areas may have few residents, but still busy streets day and night due to commerce and nightlife. If we had access to data from e.g. cellphone towers, this could have given a real-time picture of the actual size of the population at risk by detailed location. Areas with high crime counts could (and will often) be busy, and as such, the individuals’ risk of victimization need not be elevated.

Furthermore, our findings do not rule out that weather may impact the spatial distribution of crime in other contexts, e.g. in warmer climates. The significant variation in weather found in Oslo’s Nordic climate is a strong point when attempting to model weather effects. There is the possibility of a threshold effect such that the temperature relationship does not apply in cool climates, but this has not been proposed in the literature so far.

## Conclusion

In this study, we have used multivariate spatial GAM models to estimate the effect of weather on the spatial distribution of Crime in Oslo, Norway. In contrast to previous studies, also in comparable contexts, we find that no impact of multiple aspects of weather—temperature, precipitation and wind speed—on the spatial distribution of crime. The more detailed controls allowed by our modelling strategy could potentially explain that our results differ from previous findings. Future studies modelling the effect of weather on crime in other contexts using GAMs could clarify whether methodological or contextual differences are decisive.

Although it is reasonable to assume that people adjust their behavior according to weather, these findings suggest that such adjustments have very small effects on crime. For operational decisions, such as those made by the police, one should hesitate to rely on the weather forecast.

## Supplementary Information

**Additional file 1: Figure S1.** The effect of weather on the spatial distribution of property crime. Differences in predictions from models with controls for time of day and season and weather specific surfaces (Model 2) estimated for property crimes only. **Figure S2.** The effect of weather on the spatial distribution of drug related crime. Differences in predictions from models with controls for time of day and season and weather specific surfaces (Model 2) estimated for drug related crimes only. **Figure S3.** The effect of weather on the spatial distribution of violent crime. Differences in predictions from models with controls for time of day and season and weather specific surfaces (Model 2) estimated for violent crimes only. **Figure S4.** The effect of weather on the spatial distribution of crime. Differences in predictions from models without controls for time of day and season. **Figure S5.** The effect of weather on the spatial distribution of crime. Differences in predictions from models with controls for time of day and season and weather specific surfaces (Model 2), with an alternate grouping of the time variable (03:00-08:59; 09:00-14:59; 15:00-20:59; 21:00-02:59). **Table S1.** Results from Generalized Additive Models for crime counts in 100-meter grids and 6-hour slots. Separate models by crime type. The basic model includes a spatial surface (as a semiparametric smoothing spline), as well as three weather characteristics, and sets of dummies for season and time of day. Outcomes are counts of property crime, drug related crimes and violent crimes. See Figure S.2 for predicted maps. **Table S2.** Results from Generalized Additive Models for crime counts in 100-meter grids and 6-hour slots. Controls for season omitted. The outcome is grid-specific crime counts. The basic model includes a spatial surface (as a semiparametric smoothing spline), as well as three weather characteristics, and sets of dummies for time of day. In the weather specific models, the spatial surface is additionally allowed to vary with one discretized weather characteristic (rain, temperature or wind).

## Data Availability

Due to privacy concerns the data analyzed are not publicly available.

## Footnotes

1 Statistics Norway StatBank: Table 08,485. Obtained from ssb.no/statbank.

2 The crimes most affected by unknown times, e.g. theft from houses, are not driving our results. Thefts from shops etc. will be recorded within opening hours, and are unlikely to be recorded at midnight in lieu of a precise time recording.

3 The permissions for access to data did not include types of crimes where location in itself were close to identify individuals, such as violence and sexual crimes in the family, which are likely to happen at home and thus location can be enough to indirectly identify offenders and victims.

4 Our data did not contain information of more fine grained categories. A complete overview of all crime statistics codes, sorted by category, can be found here: https://www.ssb.no/klass/klassifikasjoner/146/versjon/595/koder

5 Crimes recorded outside Oslo could have geocodes outside the Oslo county, in the fjord (likely misrecordings), or in the “Marka”, or woods surrounding the city.

6 https://data.met.no/index.html

7 We had missing wind data in one time slot, and that record was deleted from all analyses.

8 In addition to the slot with the missing wind data, two slots were excluded from the first data set because they had missing values for the lagged predictors. The first observation in calendar time (00–0059 July 27^th^ 2010) by construction has no preceding observation, and hence missingness on lagged predictors. The one slot with missing wind data led to an additional observation with a missing value for wind lagged.

9 Our data use a UTM-32 projection, which is on the scale of meters. Thus, the grid structure implies that we model the data as continuous, measured with an accuracy of 100 m.

10 The variables were discretized because a three-way interaction between the spatial surface (itself a two-way interaction) and a continuous variable in a flexible specification makes for a very complex model, that did not converge. The discretization allows us to capture non-linear effects for temperature and wind speed, while retaining the parsimony required for convergence.

11 For the basic model (1), predictions are made at the median of all weather variables, setting the season to summer and the time of day to nighttime (00:00–05:59) (Fig. 1c). For interaction models predictions are made at the conditional median within each level of the factorized interaction variable (e.g., the median temperature in the third temperature tercile).

12 Days per event is calculated as 1/daily effect, where daily effect is four times the effect in a 6-h slot.
