# Supplementary material for: Blowing in the wind? Testing the effect of weather on the spatial distribution of crime using Generalized Additive Models
Source: Crime Sci. 2022 Oct 1;11(1):9. doi: 10.1186/s40163-022-00171-2 (PMC9525942; doi:10.1186/s40163-022-00171-2)
Supplement: Supplementary file 1 — Additional file 1: Figure S1. The effect of weather on the spatial distribution of property crime. Differences in predictions from models with controls for time of day and season and weather specific surfaces (Model 2) estimated for property crimes only. Figure S2. The effect of weather on the spatial distribution of drug related crime. Differences in predictions from models with controls for time of day and season and weather specific surfaces (Model 2) estimated for drug related crimes only. Figure S3. The effect of weather on the spatial distribution of violent crime. Differences in predictions from models with controls for time of day and season and weather specific surfaces (Model 2) estimated for violent crimes only. Figure S4. The effect of weather on the spatial distribution of crime. Differences in predictions from models without controls for time of day and season. Figure S5. The effect of weather on the spatial distribution of crime. Differences in predictions from models with controls for time of day and season and weather specific surfaces (Model 2), with an alternate grouping of the time variable (03:00-08:59; 09:00-14:59; 15:00-20:59; 21:00-02:59). Table S1. Results from Generalized Additive Models for crime counts in 100-meter grids and 6-hour slots. Separate models by crime type. The basic model includes a spatial surface (as a semiparametric smoothing spline), as well as three weather characteristics, and sets of dummies for season and time of day. Outcomes are counts of property crime, drug related crimes and violent crimes. See Figure S.2 for predicted maps. Table S2. Results from Generalized Additive Models for crime counts in 100-meter grids and 6-hour slots. Controls for season omitted. The outcome is grid-specific crime counts. The basic model includes a spatial surface (as a semiparametric smoothing spline), as well as three weather characteristics, and sets of dummies for time of day. In the weather specific models, the spatial surface is a [file 40163_2022_171_MOESM1_ESM.docx]

**Supplementary material**

**Figure S1: The effect of weather on the spatial distribution of property crime.** Differences in predictions from models with controls for time of day and season and weather specific surfaces (Model 2) estimated for property crimes only.

**
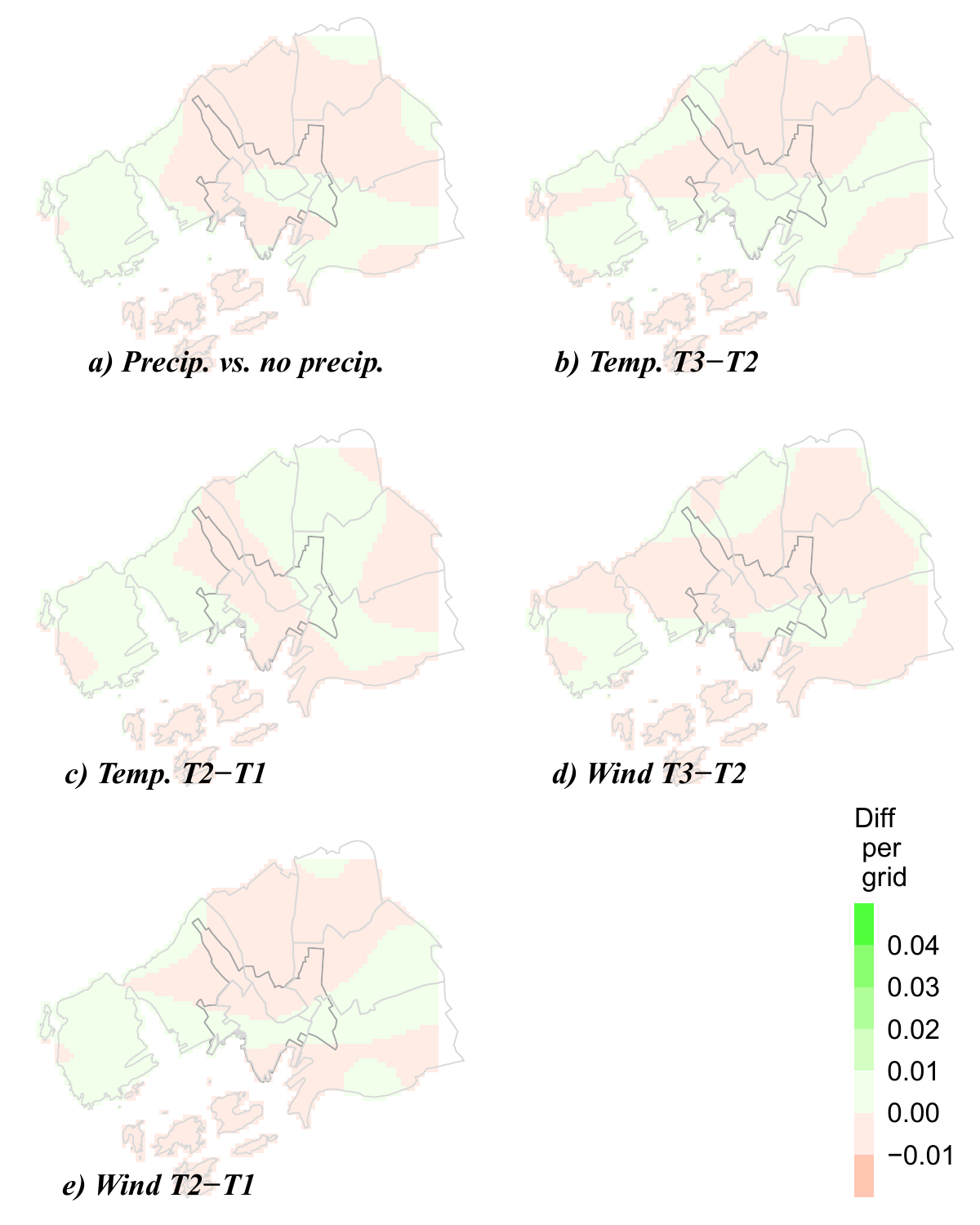
**

**Figure S2: The effect of weather on the spatial distribution of drug related crime.** Differences in predictions from models with controls for time of day and season and weather specific surfaces (Model 2) estimated for drug related crimes only.

**
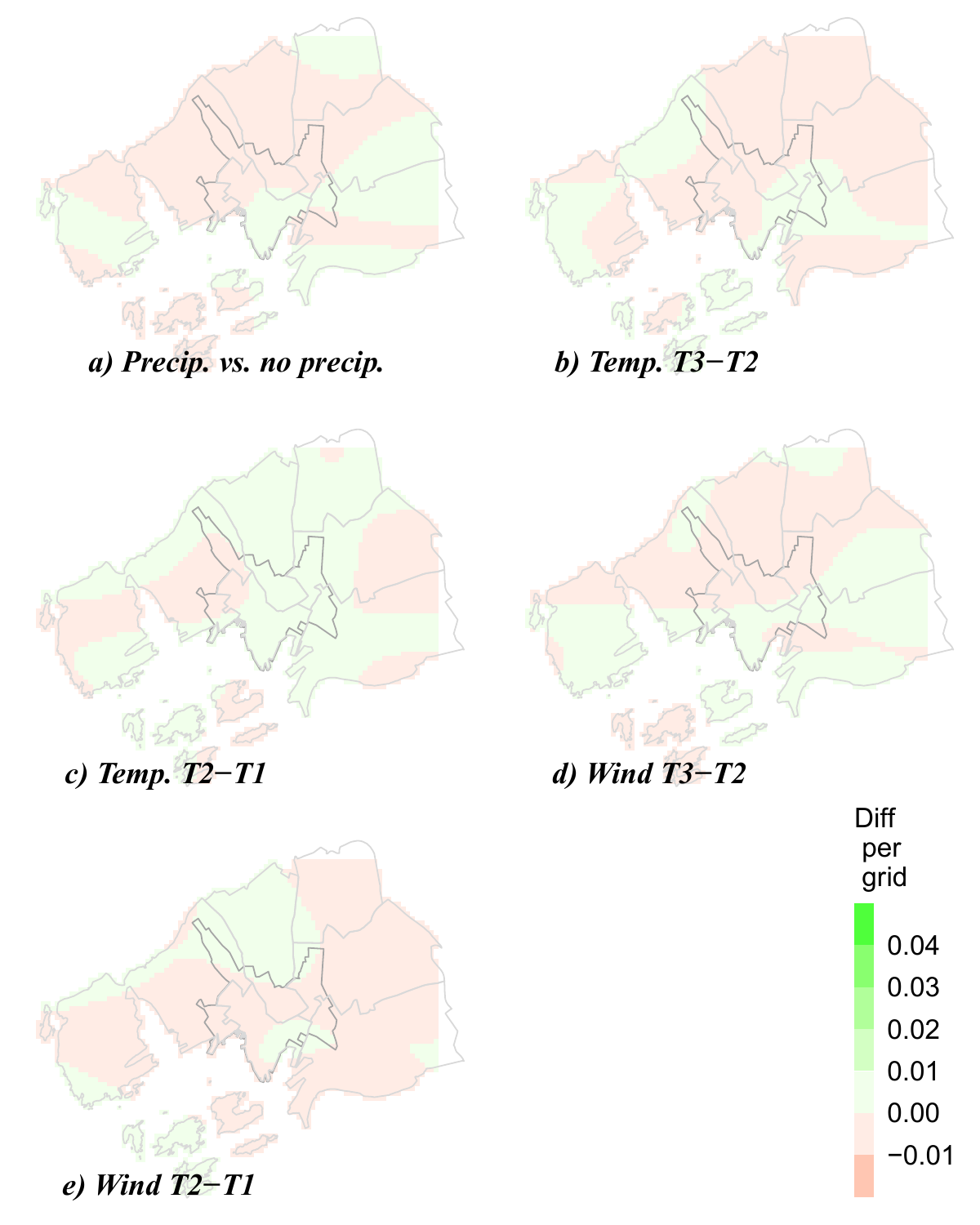
**

**Figure S3: The effect of weather on the spatial distribution of violent crime.** Differences in predictions from models with controls for time of day and season and weather specific surfaces (Model 2) estimated for violent crimes only.

**
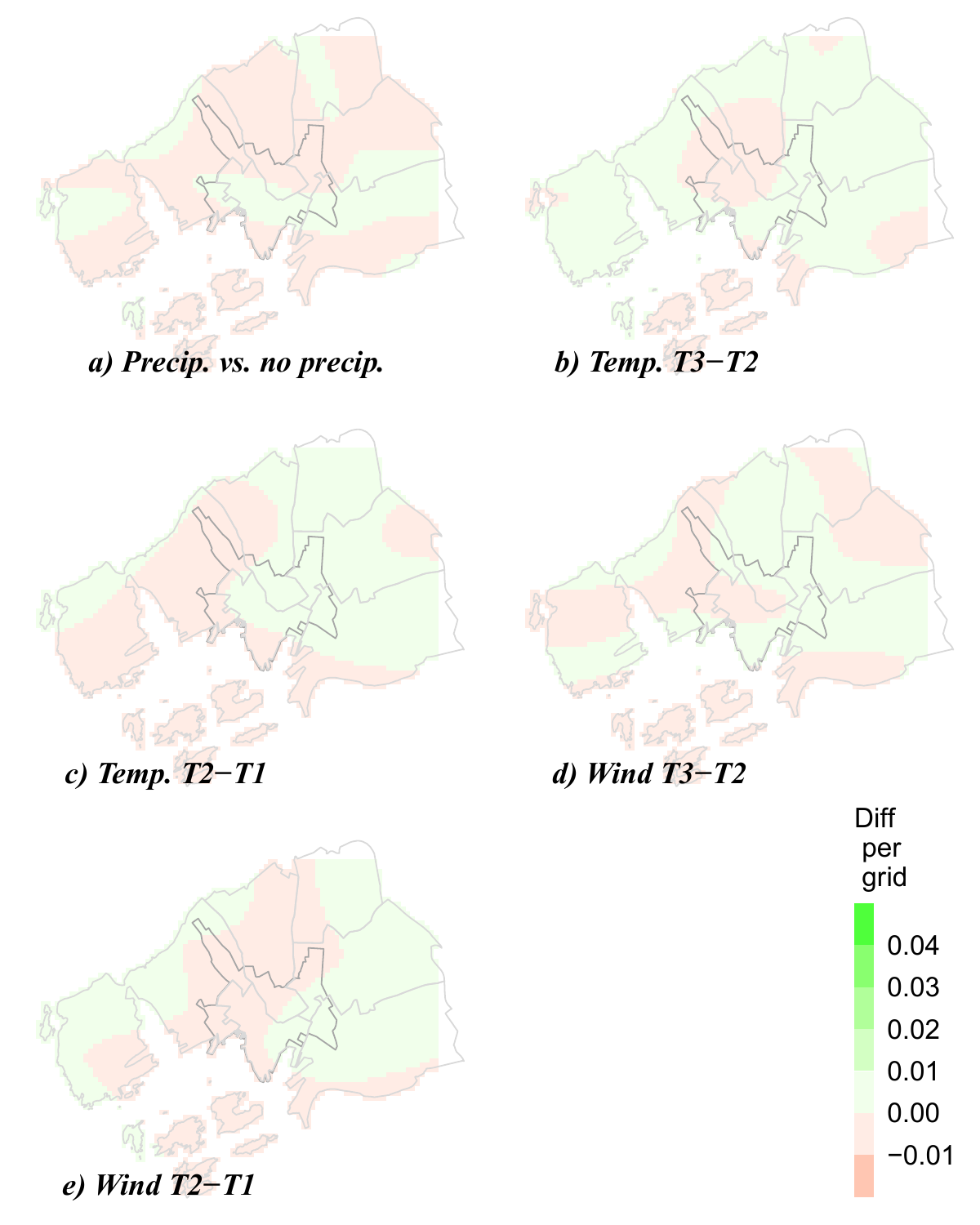
**

**Figure S4: The effect of weather on the spatial distribution of crime.** Differences in predictions from models without controls for time of day and season.


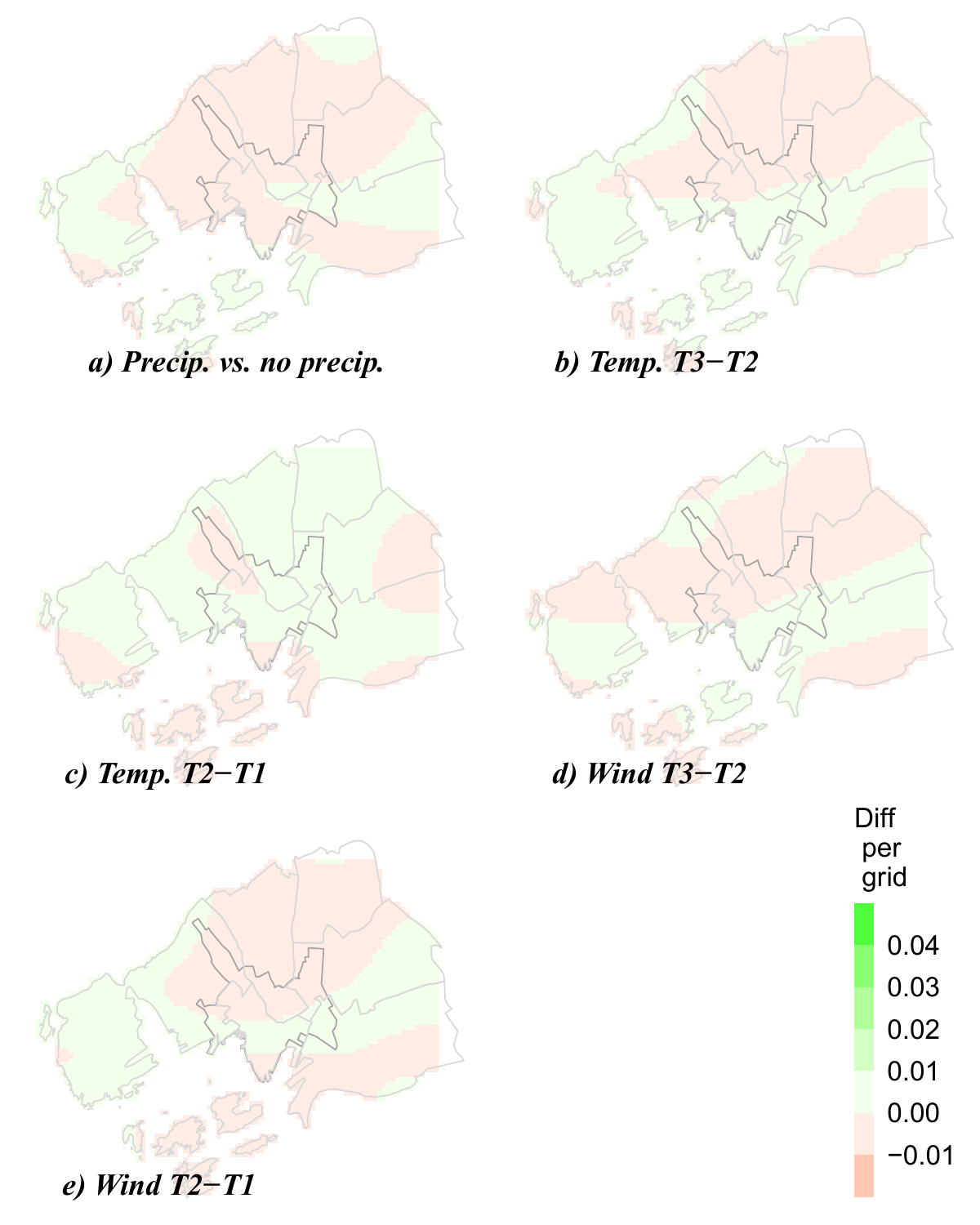


**Figure S5: The effect of weather on the spatial distribution of crime.** Differences in predictions from models with controls for time of day and season and weather specific surfaces (Model 2), with an alternate grouping of the time variable (03:00-08:59; 09:00-14:59; 15:00-20:59; 21:00-02:59).


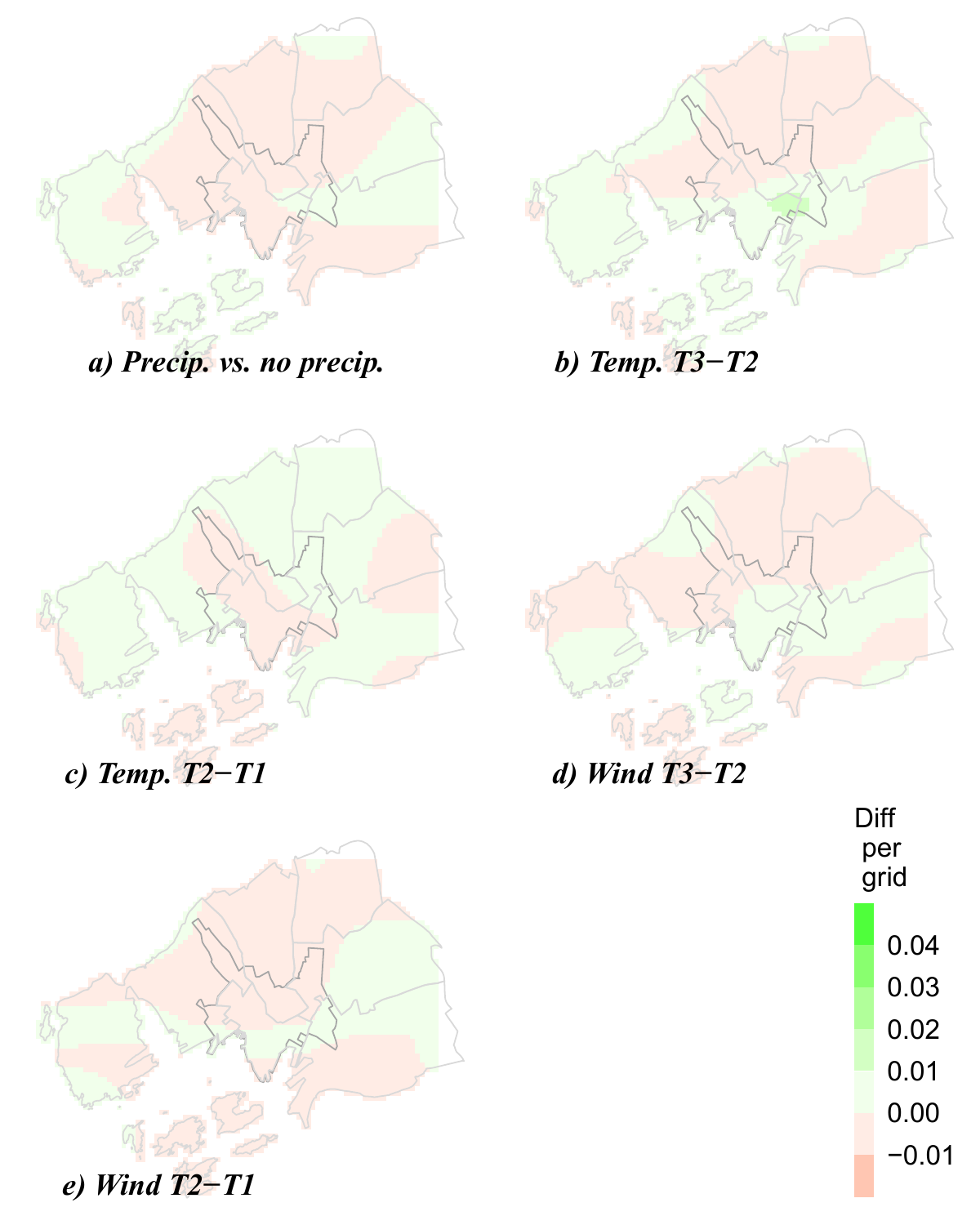


**Table S1: Results from Generalized Additive Models for crime counts in 100-meter grids and 6-hour slots. Separate models by crime type.** The basic model includes a spatial surface (as a semiparametric smoothing spline), as well as three weather characteristics, and sets of dummies for season and time of day. Outcomes are counts of property crime, drug related crimes and violent crimes. See Figure S.2 for predicted maps.

|  | **Property** | | |  | **Drugs** | | |  | **Violence** | | |  |
| --- | --- | --- | --- | --- | --- | --- | --- | --- | --- | --- | --- | --- |
| **Fixed term estimates** | **Est** | **(C.I.)** | |  | **Est** | **(C.I.)** | |  | **Est** | **(C.I.)** | |  |
| Intercept | 0,000 | (0,000- | 0,000) | *** | 0,000 | (0,000- | 0,000) | *** | 0,000 | (0,000- | 0,000) | *** |
| Temperature (degrees Celsius) | 1,001 | (1,000- | 1,002) |  | 1,005 | (1,003- | 1,008) | *** | 1,005 | (1,001- | 1,008) | ** |
| Precipitation (mm) | 0,995 | (0,992- | 0,999) | ** | 0,994 | (0,988- | 1,001) |  | 1,000 | (0,990- | 1,009) |  |
| Wind speed (m/s) | 0,995 | (0,991- | 0,998) | ** | 0,983 | (0,976- | 0,990) | *** | 1,008 | (0,997- | 1,018) |  |
| Time of day (ref=0-6) |  |  |  |  |  |  |  |  |  |  |  |  |
| 6-12 | 0,346 | (0,339- | 0,354) | *** | 0,680 | (0,648- | 0,714) | *** | 0,219 | (0,205- | 0,234) | *** |
| 12-18 | 1,000 | (0,983- | 1,018) |  | 1,699 | (1,632- | 1,768) | *** | 0,447 | (0,424- | 0,470) | *** |
| 18-24 | 0,756 | (0,743- | 0,770) | *** | 2,029 | (1,954- | 2,107) | *** | 0,522 | (0,498- | 0,547) | *** |
| Season (ref=Fall) |  |  |  |  |  |  |  |  |  |  |  |  |
| Spring | 43891,000 | (1,012- | 1,048) | ** | 1,016 | (0,980- | 1,054) |  | 0,990 | (0,942- | 1,041) |  |
| Summer | 0,927 | (0,907- | 0,947) | *** | 0,911 | (0,872- | 0,952) | *** | 0,859 | (0,809- | 0,912) | *** |
| Winter | 0,893 | (0,874- | 0,913) | *** | 0,979 | (0,936- | 1,023) |  | 0,876 | (0,824- | 0,933) | *** |
| **Smoothing splines** |  |  | EDF |  |  |  | EDF |  |  |  | **EDF** |  |
| Baseline surface |  |  | 23.93678 | *** |  |  | 23.87556 | *** |  |  | 23.43371 | *** |
| **Model fit** |  |  |  |  |  |  |  |  |  |  |  |  |
| R2 | 0,022 |  |  |  | 0,007 |  |  |  | 0,002 |  |  |  |
| Dev.expl | 0,234 |  |  |  | 0,199 |  |  |  | 0,136 |  |  |  |
| N | 20700575 | |  |  | 20547119 | |  |  | 20495967 | |  |  |

***p<.001; **p<.01; *p<.05

**Table S2: Results from Generalized Additive Models for crime counts in 100-meter grids and 6-hour slots. Controls for season omitted.** The outcome is grid-specific crime counts. The basic model includes a spatial surface (as a semiparametric smoothing spline), as well as three weather characteristics, and sets of dummies for time of day. In the weather specific models, the spatial surface is additionally allowed to vary with one discretized weather characteristic (rain, temperature or wind).

|  | **3) Basic** | | | | **4a) Precipitation** | | | | **4b) Temperature** | | |  | **4c) Wind** | | | |
| --- | --- | --- | --- | --- | --- | --- | --- | --- | --- | --- | --- | --- | --- | --- | --- | --- |
| **Fixed term estimates** | **Est** | **(C.I.)** | |  | **Est** | **(C.I.)** | |  | **Est** | **(C.I.)** | |  | **Est** | **(C.I.)** | |  |
| Intercept | 0,000 | (0,000- | 0,000) | *** | 0,000 | (0,000- | 0,000) | *** | 0,000 | (0,000- | 0,000) | *** | 0,000 | (0,000- | 0,000) | *** |
| Temperature (degrees Celsius) | 1,002 | (1,002- | 1,003) | *** | 1,002 | (1,002- | 1,003) | *** | 1,000 | (0,998- | 1,001) |  | 1,002 | (1,002- | 1,003) | *** |
| Precipitation (mm) | 0,994 | (0,992- | 0,997) | *** | 0,996 | (0,993- | 0,999) | * | 0,994 | (0,991- | 0,997) | *** | 0,994 | (0,992- | 0,997) | *** |
| Wind speed (m/s) | 0,996 | (0,993- | 0,999) | * | 0,997 | (0,994- | 1,000) | * | 0,995 | (0,992- | 0,998) | * | 0,997 | (0,991- | 1,003) |  |
| Time of day (ref=0-6) |  |  |  |  |  |  |  |  |  |  |  |  |  |  |  |  |
| 6-12 | 0,365 | (0,358- | 0,372) | *** | 0,365 | (0,358- | 0,372) | *** | 0,366 | (0,359- | 0,373) | *** | 0,365 | (0,358- | 0,372) | *** |
| 12-18 | 0,998 | (0,983- | 1,012) |  | 0,998 | (0,984- | 1,012) |  | 1,004 | (0,989- | 1,018) |  | 0,998 | (0,983- | 1,012) |  |
| 18-24 | 0,860 | (0,847- | 0,872) | *** | 0,859 | (0,847- | 0,872) | *** | 0,862 | (0,850- | 0,875) | *** | 0,860 | (0,847- | 0,872) | *** |
| **Smoothing splines** |  |  | **EDF** |  |  |  | **EDF** |  |  |  | **EDF** |  |  |  | **EDF** |  |
| Baseline surface |  |  | 23,66 | *** |  |  |  |  |  |  |  |  |  |  |  |  |
| No rain |  |  |  |  |  |  | 23,84998 | *** |  |  |  |  |  |  |  |  |
| Rain |  |  |  |  |  |  | 23,76 | *** |  |  |  |  |  |  |  |  |
| Surface T3 |  |  |  |  |  |  |  |  |  |  | 23,8445 | *** |  |  | 23,86951 | *** |
| Surface T2 |  |  |  |  |  |  |  |  |  |  | 23,81733 | *** |  |  | 23,74238 | *** |
| Surface T1 |  |  |  |  |  |  |  |  |  |  | 23,80 | *** |  |  | 23,70 | *** |
| **Model fit** |  |  |  |  |  |  |  |  |  |  |  |  |  |  |  |  |
| R2 | 0,025 |  |  |  | 0,025 |  |  |  | 0,025 |  |  |  | 0,025 |  |  |  |
| Dev.expl | 0,232 |  |  |  | 0,232 |  |  |  | 0,232 |  |  |  | 0,232 |  |  |  |
| N | 20700575 | |  |  | 20700575 | |  |  | 20700575 | |  |  | 20700575 | |  |  |

***p<.001; **p<.01; *p<.05
